# Genetic analysis of CFH and MCP in Egyptian patients with immune-complex proliferative glomerulonephritis

**DOI:** 10.3389/fimmu.2022.960068

**Published:** 2022-09-23

**Authors:** Heba R. Gouda, Iman M. Talaat, Amal Bouzid, Hoda El-Assi, Amira Nabil, Thenmozhi Venkatachalam, Poorna Manasa Bhamidimarri, Inken Wohlers, Amena Mahdami, Saba EL-Gendi, Ahmed ElKoraie, Hauke Busch, Maha Saber-Ayad, Rifat Hamoudi, Nahed Baddour

**Affiliations:** ^1^ Pathology Department, Faculty of Medicine, Alexandria University, Alexandria, Egypt; ^2^ Clinical Sciences Department, College of Medicine, University of Sharjah, Sharjah, United Arab Emirates; ^3^ Sharjah Institute for Medical Research, University of Sharjah, Sharjah, United Arab Emirates; ^4^ Human Genetics Unit, Pathology Department, Faculty of Medicine, Alexandria University, Alexandria, Egypt; ^5^ Human Genetics Department, Medical Research Institute, Alexandria University, Alexandria, Egypt; ^6^ Department of Physiology and Immunology, College of Medicine, Khalifa University, Abu Dhabi, United Arab Emirates; ^7^ Medical Systems Biology Division, Lübeck Institute of Experimental Dermatology and Institute for Cardiogenetics, University of Lübeck, Lübeck, Germany; ^8^ Nephrology Unit, Internal Medicine Department, Faculty of Medicine, Alexandria University, Alexandria, Egypt; ^9^ Pharmacology Department, Faculty of Medicine, Cairo University, Cairo, Egypt; ^10^ Division of Surgery and Interventional Science, University College London, London, United Kingdom

**Keywords:** complement system, glomerulonephritis, CFH, MCP, lupus nephritis, post-infectious glomerulonephritis

## Abstract

Glomerulonephritis (GN) is a complex disease with intricate underlying pathogenic mechanisms. The possible role of underlying complement dysregulation is not fully elucidated in some GN subsets, especially in the setting of autoimmunity or infection. In the current study, diagnosed cases of lupus nephritis (LN) and post-infectious GN (PIGN) were recruited for molecular genetic analysis and targeted next-generation DNA sequencing was performed for two main complement regulating genes: in the fluid phase; *CFH*, and on tissue surfaces; *MCP*. Three heterozygous pathogenic variants in *CFH* (Q172*, W701*, and W1096*) and one likely pathogenic heterozygous variant in *MCP* (C223R) have been identified in four of the studied LN cases. Additionally, among the several detected variants of uncertain significance, one novel variant (CFH:F614S) was identified in 74% of the studied LN cases and in 65% of the studied PIGN cases. This variant was detected for the first time in the Egyptian population. These findings suggest that subtle mutations may be present in complement regulating genes in patients with immune-complex mediated category of GN that may add to the disease pathogenesis. These findings also call for further studies to delineate the impact of these gene variants on the protein function, the disease course, and outcome.

## Introduction

Immune complex-mediated glomerulonephritis (ICGN) is the most common form of proliferative glomerulonephritis (GN) (1). Infections and autoimmune diseases such as lupus nephritis (LN) are common causes of ICGN in Egypt (2, 3). Deposition of immune complexes in the glomeruli activates the complement system through the classical pathway which is the major pathogenic driver of ICGN. Several observations have led to the assumption of an associated role for underlying dysregulation of the alternative pathway (4). Enhanced activity of the alternative pathway, either due to genetic or acquired causes, may be one of the factors augmenting tissue injury (5). Genetic variants in complement regulating genes have been detected in association with most types of ICGN. Examples include post-infectious glomerulonephritis (PIGN) (6, 7), autoimmune diseases such as LN (8, 9), and IgA nephropathy (10–12). Because GN is a significant cause of chronic kidney disease that may eventually require costly renal replacement therapy, understanding the underlying pathogenic causes is of utmost importance and may guide revealing new targets for therapy (13).

A group of regulators and inhibitors tightly controls the complement system to prevent unchecked activation and undesired tissue injury. These regulators are circulating in the plasma or on cell surfaces (14). Factor H (FH) is the main regulator of alternative pathway activity. It is a fluid phase regulator, synthesized in the liver and found soluble in plasma (15). The main function of FH is to distinguish self-antigens from non-self and to prevent complement activation on host cells (16). The FH protein is composed of 20 short consensus repeat (SCR) domains each comprising about 60 amino acids. The gene encoding for FH is Complement Factor H gene (*CFH*), located on the long arm of chromosome 1. It comprises 23 exons and spans over 94 kb (17, 18). Mutations in the CFH gene are associated with several diseases such as atypical hemolytic uremic syndrome (aHUS), dense deposit disease (DDD), and age-related macular degeneration (19, 20). Deficiency of FH was found to accelerate the development of LN and was associated with clinical and pathologic activities in patients with LN (21, 22).

Membrane cofactor protein (MCP), also known as CD46, is a transmembrane glycoprotein, widely expressed in almost all human tissues. It controls complement activation on cell surfaces. The mature protein consists of four SCR domains that house the sites for regulatory activity, followed by an alternatively spliced region for O-glycosylation, a juxta membranous domain, a hydrophobic transmembrane domain, and one of two alternatively spliced charged cytoplasmic tails. The gene encoding for CD46 is located on the long arm of chromosome 1 and consists of 14 exons spanning over 43 kb. Mutations in the CD46 gene have been implicated in many glomerular diseases, especially aHUS, pregnancy-related disorders, and LN. Most mutations occur in the four SCRs regions (23).

Lupus nephritis is one of the common causes of ICGN. Up to 50% of patients with systemic lupus erythematosus (SLE) have clinically evident kidney disease at the time of presentation, and up to 75% develop it during the disease (24). It is characterized by the formation of immune complexes that deposit in the kidneys together with complement components. Complement activation in LN primarily occurs through the classical pathway that mediates glomerular injury *via* the production of chemotactic factors that recruit neutrophils and monocytes, with the generation of membrane attack complex (MAC) (25). The role of the complement system in the pathogenesis of LN has long been poorly understood. Although hereditary deficiency of early complement components such as C1, C4, and C2 is one of the risk factors that predispose for SLE (26), complement overactivation due to dysregulation of the alternative pathway has also been found to accelerate the disease progression and enhance the development of nephritis both in animal models and in humans (21, 22, 26–31).

Another common cause of ICGN frequently encountered among Egyptian GN patients is the post-infectious GN (PIGN) that causes acute nephritic syndrome in children (32). Although its pathogenesis is classically described as an immune complex-mediated disease, underlying genetic dysregulation of the alternative complement pathway has been noticed, especially in patients with an atypical or prolonged course of PIGN (6, 33–36). Mutations in genes encoding for regulators of the alternative pathway of complement activation, such as FH or factor H-related protein-5 (CFHR5), have been reported in some cases of PIGN (6, 7).

This study aimed to explore the genetic variations in *CFH* and *CD46* genes among a cohort of Egyptian patients with ICGN, including a group of children with PIGN and a group of LN patients.

## Materials and methods

Forty well-characterized renal core biopsy specimens from the archives of Pathology Laboratory, Alexandria Faculty of Medicine, were included in the study. The study was conducted in accordance with the Declaration of Helsinki and approved by the Ethics Committee of the Faculty of Medicine, Alexandria University (serial No. 0201148, IRB No. 00012098, FWA No. 00018699 in September 2018). Twenty-three cases were diagnosed as LN, and seventeen were diagnosed as PIGN. All SLE patients included in the study fulfilled the American College of Rheumatology diagnostic criteria for the SLE (37). All cases with PIGN had a preceding history of infection, elevated anti-streptolysin O titre and renal biopsy confirmed the diagnosis. Twenty-two studied cases were children (<18 years), and 18 were adults.

All cases met the following inclusion criteria; 1) presence of any pattern of proliferative glomerulonephritis as seen by light microscopic examination; whether mesangial hypercellularity, endocapillary or extracapillary hypercellularity, 2) positive immunohistochemical staining for immunoglobulins (IgG and/or IgM, and/or IgA), and C3, 3) availability of clinical data or laboratory investigations confirming the underlying etiology of ICGN. LN patients with pure class V disease were not included due to a lack of proliferative lesions. Patients with clinical, laboratory or histopathologic suspicion of the hemolytic uremic syndrome and biopsies with sole deposition of C3 were excluded from the study.

### Collection of clinical data and laboratory investigations

Clinical information such as age, sex, and family history of kidney disease, as well as the laboratory investigation such as serum creatinine level, urine analysis for proteinuria and/or hematuria, serum complement levels (measured by nephelometry using BN prospec device (Siemens Health Care Diagnostics, USA)), and immunology profile, were obtained from the Nephrology units, Alexandria University Hospital, Egypt.

### Histopathologic examination of kidney biopsies

Renal tissues embedded in paraffin were cut into 3 to 4 microns thick sections and were stained with hematoxylin-eosin, Masson’s trichrome and Periodic Acid-Schiff. Two independent pathologists evaluated the biopsies. All post-infectious GN biopsies were assessed for mesangial hypercellularity, endocapillary hypercellularity, neutrophil exudation, crescent formation, fibrinoid necrosis, interstitial inflammation, globally sclerotic glomeruli, the extent of interstitial fibrosis and tubular atrophy and presence or absence of chronic vascular changes. Kidney biopsies of LN cases were classified according to the international society of nephrology/renal pathology society (ISN/RPS) system (38, 39). Activity and chronicity indices were scored for all LN cases according to the modified National Institute of Health (NIH) scoring system (38).

Immunohistochemical staining for IgG, IgA, IgM, and C3 was done on all biopsies using 4 microns thick deparaffinized and rehydrated sections of formalin-fixed paraffin-embedded (FFPE) renal tissues using ready to use rabbit polyclonal antibodies against IgG, IgA, IgM, and C3 (Cell Marque, USA, catalogue numbers; 269A-18, 267A-18, 270A-18, and 403A-78 respectively). The staining pattern was semi-quantitatively graded from 0 to 3+ (40).

### DNA extraction from tissue biopsy

Genomic DNA was extracted from curls obtained from FFPE blocks using a QIAamp DNA tissue kit (Qiagen, Germany) according to the manufacturer’s instructions (41). Based on the amount of tissue, 3 curls from the tissue blocks were taken and deparaffinized with Xylene and ethanol followed by lysis with Proteinase K and ATL tissue lysis buffer at 56°C for 2.5 hours. The digested samples were then treated with AL buffer and 100% ethanol and washed with wash buffers and eluted in 30uL nuclease-free water. The quantity and the quality of the extracted DNA were analyzed by Nanodrop. The average amount of DNA used for the mutational screening is around 5ng.

### Primer designing and validation

Multiplex primers were designed to cover complete exonic regions of *CFH* (22 coding exons) and *CD46* (13 coding exons), using the Primer3 tool. The details of the primers used in the study for *CFH*, *CD46* are listed in **Supplementary Tables S1, S2**, respectively. The primers were then evaluated using control DNA samples and the amplicon sizes were analyzed on 2% agarose gel (MBG, Bio Basic Inc.). Accession numbers for the genes: *CFH:* NM_000186.4 and *CD46*: NM_172359.3.

### Targeted DNA sequencing using Fluidigm access array

The validated target-specific primers were then attached with Fluidigm specific tag sequences and used for a targeted next-generation sequencing as previously described (42, 43). Briefly, a minimum of 5ng of DNA was taken from each sample and was amplified with 10uM of each tagged target-specific primer using Fast Start High Fidelity master mix (Roche, Switzerland) with the following cycling conditions; 1 cycle of 95°C 10 min, 2 cycles of 95°C 15 sec, 60°C 4 min and 13 cycles of 95°C 15 sec, 72°C 4 min. The amplified products were further purified using ExoSAP-IT (Invitrogen, USA) and then diluted with Nuclease free water. The purified amplicons were then amplified with the Tagged target-specific primers on the 48.48 Access Array integrated fluidic circuit (IFC) using a Fast start high fidelity master mix (Roche, Switzerland). The PCR products were then harvested from the 48.48 Fluidigm Access Array IFC (Fluidigm Europe B.V, Netherlands) and carefully transferred from each of the sample inlets and linked with 48.48 Access Array IFC barcodes. The prepared amplicon library was purified using AMPure XP beads (Beckman Coulter, USA) and quantified using High sensitivity DNA assay kit on BioAnalyzer (Agilent, USA). The libraries were further diluted to 1pg and were amplified using emulsion PCR with Ion SphereTM particles with Ion Template OT2 kit (Ion OneTouch™ instrument) in Ion OneTouch™ ES system following manufacturer’s instructions (Thermo Fischer, USA). The pooled samples were then sequenced using the Ion 520™ Chip on the Ion S5 XL Semiconductor sequencer following the manufacturer’s instructions (Thermo Fisher).

### Variant analyses and interpretation

All samples underwent quality control analysis according to the guidelines of College of American Pathologists (44). Sequenced reads were aligned to the reference human genome NCBI37 (hg19) by burrows wheeler aligner (BWA) on default settings (45). Binary alignment map (BAM) files were generated using Ion torrent suite version 5.12.3. Single nucleotide variants (SNVs) and small insertion/deletion events (indels) were identified by SAMtools. Reads were visualized using integrative genomics viewer (IGV) (46), with the appropriate browser extensible data (BED) files for both genes. Genomic positions failing to meet a coverage depth of at least 50X, or those failing to meet a variant quality call of 30, were filtered out of the downstream analysis. Candidate variants were filtered based on a population MAF of 0.1%; this cut-off was chosen because it was found that complement gene variants with a MAF <0.1% are of interest in terms of complement dysregulation (47). Rare variants were then analyzed and scored for predicted pathogenicity as pathogenic, likely pathogenic, uncertain significance (VUS), likely benign, or benign based on the scheme outlined by the American College of Medical Genetics and Genomics and association of molecular pathology (ACMG/AMP) (48). Variants of uncertain significance were classified based on a prediction by *in-silico* tools PolyPhen-2, SIFT, and MutationTaster.

### Sanger validation of the variant CFH:c.1841T>C (F614S)

Samples with a good amount of DNA were selected to validate the variant identified on NGS for the *CFH* gene at the position 196694395 (T/C). Primers for the flanking sequences of the SNP were designed using Primer3 BLAST. The target region was amplified using forward: 5’ AAGAAAGAATGCGAACTTCC 3’ and reverse: 5’ GGAGGTCAGGAGACAATCCA 3’ primers. The sequencing reactions were loaded on the ABI 3730xl sequencer (Applied Biosystems). Sequencing data were aligned to the genomic reference sequence and analyzed using BioLign software (version 4.0.6.2) software.

### Review of genetic variation in ancestry-matched reference data

Genetic variation in the *CFH* and *CD46* genes was reviewed in an ancestry-matched reference cohort of overall 110 Egyptian individuals. Towards this, variant calling performed by Wohlers et al. (49) was used (EGA data set EGAD00001006039). This variant calling is based on largely low-coverage Illumina short-read sequencing of 100 individuals obtained from Pagani et al. (50) (EGA data sets EGAD00001001372 and EGAD00001001380) as well as high-coverage Illumina short-read sequencing of 10 individuals performed by Wohlers et al. (49) (EGA data set EGAD00001006037). Single nucleotide variants within +/- 100 kb and SVs within 10 Mb of gene boundaries according to Ensembl were extracted and inspected *via* IGV. For this, GRCh37 coordinates of the novel variants identified in this study were lifted to GRCh38 using the online version of UCSC’s LiftOver tool (https://genome.ucsc.edu/cgi-bin/hgLiftOver).

## Results

### Clinical characteristics

The study included 23 cases of LN and 17 cases of PIGN. The main clinical characteristics and laboratory investigations of the patients at the time of biopsy are shown in **Table 1**.

**Table 1:** Summary of the clinical characteristics and laboratory investigations of the 40 studied cases.

		Whole cohort		LN group		PIGN group	
		Number (n = 40)	%	Number (n = 23)	%	Number (n = 17)	%
**Age (years)**	Child (> 18)	22	55	5	21	17	100
	Adult (≥ 18)	18	45	18	79	0	0
	Median (IQR) (years)	14 (11-25)		23 (18-32)		11 (7-12.5)	
**Sex**	Male	20	50	6	26	14	82
	Female	20	50	17	74	3	18
**Proteinuria** **(g/day)**	Median (IQR)	1.8 (1.3-2.4)		2 (1.5-2.5)		1.5 (0.9-2)	
**Serum creatinine level (mg/dl)**	Median (IQR)	1.2 (0.9-2.5)		1 (0.9-2)		1.5 (1-2.9)	
**Serum C3 level**	low (<90mg/dl)	31	77.5	14	61	17	100
	Normal	9	22.5	9	39	0	0
**Serum C4 level**	low (<10mg/dl)	40	100	23	100	17	100
	Normal	0	0	0	0	0	0

LN, lupus nephritis; PIGN, post-infectious glomerulonephritis. Normal serum creatinine 0.7-1.3 mg/dl, normal serum C3 90-180 mg/dl, normal serum C4 10-45mg/dl.

### Histopathologic findings

All biopsies showed variable degrees of cellular proliferation in the glomeruli as well as evident immune complex and C3 deposits as per inclusion criteria. None of the cases had evidence of arterial mucoid intimal oedema, fibrin thrombi or necrotizing vasculitis (excluding possibly associated thrombotic microangiopathy). Cases with sole C3 deposition were not included in the study (excluding C3 glomerulopathy (DDD or C3GN)). A Summary of the histopathologic and immunohistochemical findings is shown in **Table 2**. Detailed clinical and histopathologic findings of LN and PIGN cases are shown in **Supplementary Tables S3, S4**, respectively.

**Table 2:** Summary of the histopathologic and immunohistochemical findings of the 40 studied cases.

			AI (range/24)	CI (range/12)	Immunostaining (0-3+)			
					IgG	IgA	IgM	C3
**Lupus Nephritis** **(n=23)**	Class II	9	0-1	0-2	2-3	0-1	0-1	2-3
	Class III	5	2-8	0-2	2-3	0-1	0-1	2-3
	Class IV	7	8-11	0-4	3	1-2	0-2	2-3
	Class III+V	1	8	3	3	1	0	3
	Class IV+V	1	10	4	3	0	0	3
**PIGN** **(n=17)**					1-3	0	0-1	2-3

AI, Activity index according to the modified national institute of health scoring system (38). CI, Chronicity index according to the modified national institute of health scoring system (38). PIGN, post-infectious glomerulonephritis.

### Targeted next-generation sequencing results

DNA samples from the 40 patients were tested for genetic variants in CFH and CD46 genes. Thirty-seven rare variants (minor allele frequency (MAF) in general population <0.1%) were identified in 32 patients (80%); 29 variants were found in CFH gene, and eight variants were detected in CD46 gene. Eleven (29.7%) out of the identified 37 rare variants were classified as benign or likely benign according to the American College of Medical Genetics and Genomics (ACMG) guidelines (48). Only one of these benign variants (CFH: c.1652T>C, p.I551T) was found on the Egyptian genome reference (EgyptRef) (49) with an allele frequency (AF) of 0.009. These 11 variants were excluded from being of interest as they are most likely not causally related to the disease process. A list of the detected benign variants is shown in **Table 3**. Allele frequencies of Benign/Likely benign variants in different populations are shown in **Supplementary Table S5**.

**Table 3:** Benign/Likely benign variants.

Gene	Patient	Chromosomal position*		AA change	Nucleotide change	Exon	Type	Zygosity	dbSNP ID	AF (EgyptRef)	Global AF
***CFH***	PI17	196643015		p.T91=	c.273T>C	Exon 3	Synonymous	Het	–	–	–
***CFH***	LN16	196646739		p.D187=	c.561T>C	Exon 5	Synonymous	Het	–	–	–
***CFH***	LN3	196654300		p.Y299=	c.897T>C	Exon 7	Synonymous	Het	–	–	–
***CFH***	LN14	196684820		p.G539=	c.1617T>C	Exon 12	Synonymous	Het	rs147170171	–	0.0000598
***CFH***	PI19	196684844		p.T547=	c.1641C>T	Exon 12	Synonymous	Het	–	–	–
***CFH***	LN3	196684855		p.I551T	c.1652T>C	Exon 12	Missense	Het	rs35453854	0.009091	0.00396
***CFH***	PI18	196684859		p.V552=	c.1656G>A	Exon 12	Synonymous	Hom	–	–	–
***CFH***	PI20	196697588		p.G783=	c.2349A>T	Exon 16	Synonymous	Het	–	–	–
***CFH***	PI11	196712655		p.S1096=	c.3207T>C	Exon 21	Synonymous	Het	rs62641697	–	0.00104
***CD46***	PI21	207925593		p.P12=	c.36T>C	Exon 1	Synonymous	Het	–	–	–
***CD46***	LN 18	207925626		p.A23=	c.69C>T	Exon 1	Synonymous	Het	–	–	–

PI, post-infectious; LN, lupus nephritis; Het, heterozygous; Hom, homozygous; AF, Global (gnomAD: Exomes).

*Chromosomal positions are according to GRCh37.

Twenty-two (59.5%) out of the 37 rare variants were classified as variants of uncertain significance (VUS) according to the ACMG guidelines (48). These were detected in 31 patients. None of these variants was found on the Egyptian genome reference (EgyptRef). Most were heterozygous except for four variants that were found in a homozygous state. After analysis by *in-silico* tools (PolyPhen-2, SIFT, and MutationTaster), variants of uncertain significance were either found benign in all used tools (10 variants), or had mixed results (6 variants), or were found to be pathogenic in all used tools (1 variant). Five variants were noncoding intronic variants so *in-silico* prediction by PolyPhen-2, SIFT and MutationTaster was not applicable. Each of the identified VUS was only detected once i.e. in one patient, except for the variant (*CFH*:F614S) that was found in 28 (70%) of patients: 17 (74%) of LN patients, and 11 (65%) of PIGN patients, and was predicted to be pathogenic by all used *in-silico* tools. It is a missense variant located at position 196694395 in exon 13 of *CFH* and results in phenylalanine to serine substitution on position 614 of the protein (SCR10). Variants of uncertain significance are summarized in **Table 4**. Allele Frequencies of Variants of uncertain in different populations are shown in **Supplementary Table S6**.

**Table 4:** Variants of uncertain significance are classified according to the *in-silico* prediction tools.

Gene	Patient	Chromosomal position*		AA change	Nucleotide change	Exon/Intron	Type	Zygosity	dbSNP ID	AF (EgyptRef)		AF
**Variants of uncertain significance predicted as benign, tolerated or polymorphism on PolyPhen-2, SIFT and MutationTaster**												
***CFH***	PI21		196642242	V65I	c.193G>A	Exon 2	Missense	Het	rs747978546	**-**	0.0000359	
***CFH***	LN2		196643037	T99A	c.295A>G	Exon 3	Missense	Hom	–	–	–	
***CFH***	LN19		196695675	G650E	c.1949G>A	Exon 14	Missense	Het	–	–	–	
***CFH***	PI14		196695705	N660S	c.1979A>G	Exon 14	Missense	Het	–	–	–	
***CFH***	LN19		196695720	M665T	c.1994T>C	Exon 14	Missense	Het	–	–	–	
***CFH***	PI17		196696016	H278Y	c.2182C>T	Exon 15	Missense	Het	–	–	–	
***CFH***	PI22		196697572	R778T	c.2333G>C	Exon 16	Missense	Het	rs767471170	*-*	0.00000399	
***CFH***	LN18		196706002	H821R	c.2462A>G	Exon 17	Missense	Het	–	–	–	
***CFH***	PI1		196709801	M945I	c.2835G>A	Exon 19	Missense	Hom	–	–	–	
***CD46***	LN18		207958447	A353=	c.1059C>T	Exon 12	Synonymous	Het	–	–	–	
**Variants of uncertain significance having mixed prediction results on PolyPhen-2, SIFT and MutationTaster**												
***CFH***	LN17		196684824	E541K	c.1621G>A	Exon 12	Missense	Hom	rs865963138	–	–	
***CFH***	LN2		196684854	I551V	c.1651A>G	Exon 12	Missense	Het	–	–	–	
***CFH***	PI1		196711088	V1014M	c.3040G>A	Exon 20	Missense	Het	–	–	–	
***CD46***	PI10		207925585	P10S	c.28C>T	Exon1	Missense	Hom	–	–	–	
***CD46***	LN2		207925621	A22T	c.64G>A	Exon 1	Missense	Het	–	–	–	
***CD46***	LN17		207940529	K282R	c.845A>G	Exon 6	Missense	Het	–	–	–	
**Variant of uncertain significance predicted as damaging, deleterious or disease-causing on PolyPhen-2, SIFT and MutationTaster**												
***CFH***	28 patients		196694395	F614S	c.1841T>C	Exon 13	Missense	Het (in all but one case)	Novel	–	–	
**Intronic noncoding variants**												
***CFH***	LN4		196621191	–	c.-57G>A	Intron 1	5’UTR	Het	–	–	–	
***CFH***	LN15		196621330	–	c.58+25T>C	Intron 1	Intronic variant	Het	–	–	–	
***CFH***	LN21		196645080	–	c.351-39A>G	Intron 3	Intronic variant	Het	–	–	–	
***CFH***	LN2		196658757	–	c.1159+13C>T	Intron 8	Intronic variant	Het	–	–	–	
***CD46***	LN18		207932924	–	c.390-60T>C	Intron 3	Intronic variant	Het	–	–	–	

PI, post-infectious; LN, lupus nephritis; Het, heterozygous; Hom, homozygous. EgyptRef, Egyptian Genome Reference; AF, Global (gnomAD: Exomes). *Chromosomal positions are according to GRCh37.

Three pathogenic mutations CFH:c.514C>T (p.Q172*, SCR3) in exon 5, CFH:c.2103G>A (p.W701*, SCR12) in exon 15, and CFH:c3288G>A (p.W1096*, SCR18) in exon 21, and one likely pathogenic mutation CD46:c.667T>C (p.C223R, SCR3) were detected in our cohort. All four mutations were heterozygous and were detected in LN patients. No pathogenic or likely pathogenic mutation was detected in any of the cases of PIGN. Pathogenic and likely pathogenic variants are summarized in **Table 5**. Detailed clinical and histologic findings in cases with pathogenic or likely pathogenic variants are detailed in **Supplementary Table S3**.

**Table 5:** Pathogenic or likely pathogenic variants.

Gene	Patient	LN class	Chromosomal position*	AA change	SCR	Nucleotide change	Exon	Type	Zygosity	ACMG lassification	dbSNP ID	AF (EgyptRef)	AF
***CFH***	LN12	IV	196646692	Q172*	SCR3	c.514C>T	Exon 5	Nonsense	Het	Pathogenic	–	–	–
***CFH***	LN7	III+V	196695937	W701*	SCR12	c.2103G>A	Exon 15	Nonsense	Het	Pathogenic	–	–	–
***CFH***	LN19	III	196712736	W1096*	SCR18	c.3288G>A	Exon 21	Nonsense	Het	Pathogenic	–	–	–
***CD46***	LN6	IV+V	207934785	C223R	SCR3	c.667T>C	Exon 5	Missense	Het	Likely pathogenic	–	–	–

LN, lupus nephritis; Het,heterozygous; EgyptRef, Egyptian Genome Reference; AF, Global (gnomAD: Exomes).

*Chromosomal positions are according to GRCh37.

**Figure 1:**
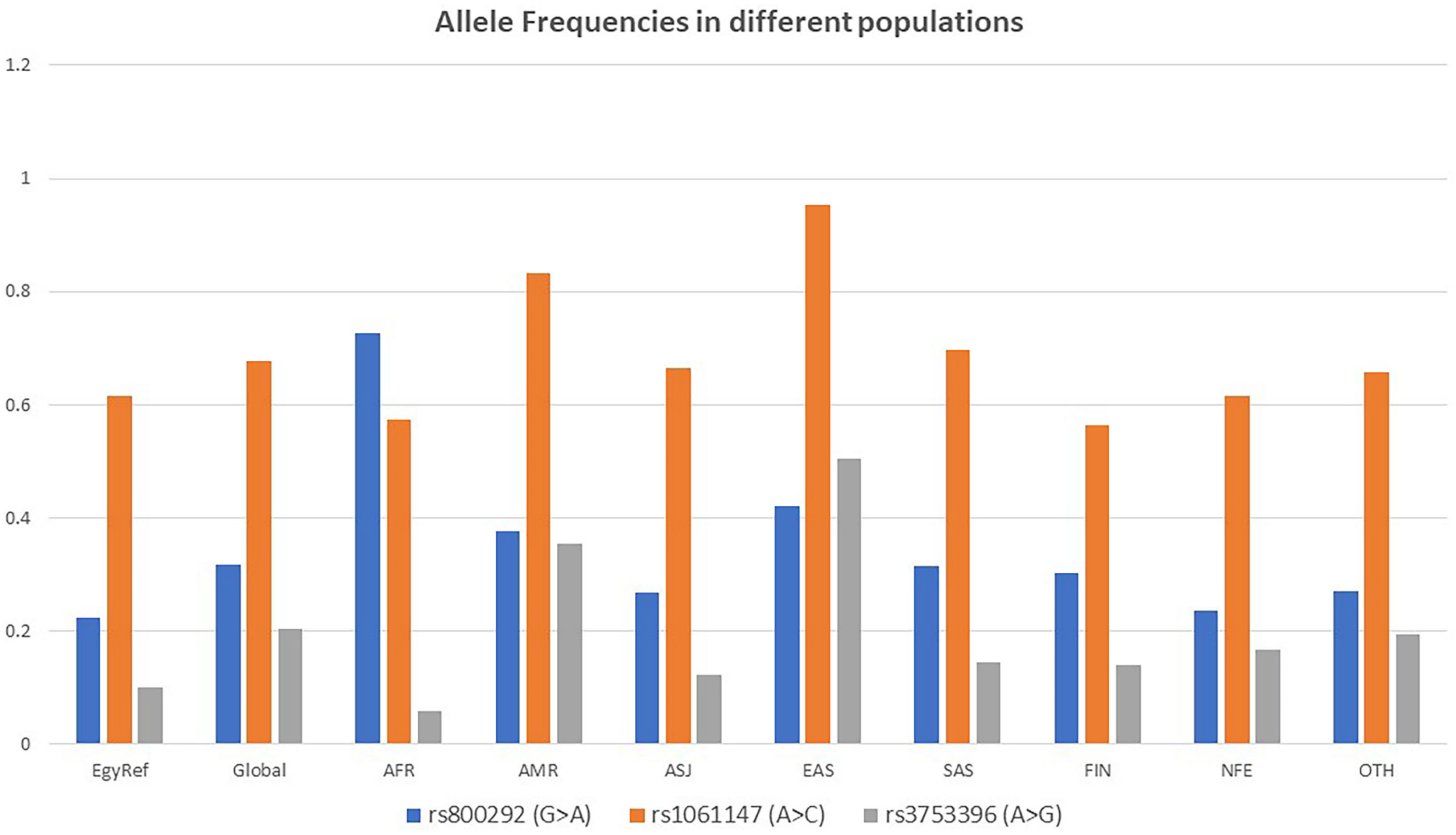
Allele Frequencies of the detected recurrent SNPs in different populations according to gnomAD. EgyptRef, Egyptian Genome Reference; AF, Global (gnomAD: Exomes); AFR, African; AMR, American; ASJ, Ashkenazi Jewish; EAS, East Asian; SAS, South Asian; FIN, European (Finnish); NFE, European (Non-Finish); OTH, Other.

Three single nucleotide polymorphisms (SNPs) in *CFH* gene were detected in the current study and were found to be commonly encountered in the population according to the 1000 Genomes project (51) and gnomAD (52). These are rs800292, rs1061147, and rs3753396. All were predicted to be benign variants. The SNP rs800292 was detected in 13 (32.5%) out of 40 patients (6 (26%) of LN patients, and 7 (41%) of post-infectious GN patients). The SNP rs1061147 was detected in 5 (12.5%) out of 40 patients, all had LN (21.7% of LN patients). The SNP rs3753396 was detected in 8 (20%) out of 40 patients (5 (21.7%) of LN patients, and 3 (17.6%) of post-infectious GN patients). A summary of the recurrent SNPs in the studied cases is shown in **Table 6**. Allele Frequencies of the detected recurrent SNPs in different populations are shown in **Figure 1**.

**Table 6:** Summary of benign common SNPs.

Gene	Number of patients	Exon	Domain	Nucleotide change	Chromosomal position*	Variant (coding)	Type	dbSNP ID	AF(EgyptRef)	AF
***CFH***	13	2	SCR1	c.184G>A	196642233	V62I	Missense	rs800292	0.223	0.317
***CFH***	5	7	SCR5	c.921A>C	196654324	A307=	Synonymous	rs1061147	0.615	0.676
***CFH***	8	14	SCR11	c.2016A>G	196695742	Q672=	Synonymous	rs3753396	0.101	0.204

EgyptRef, Egyptian Genome Reference; AF, Global (gnomAD: Exomes).

*Chromosomal positions are according to GRCh37.

### Sanger sequencing to confirm the presence of the novel variant CFH:c.1841T>C (F614S)

**Figure 2:**
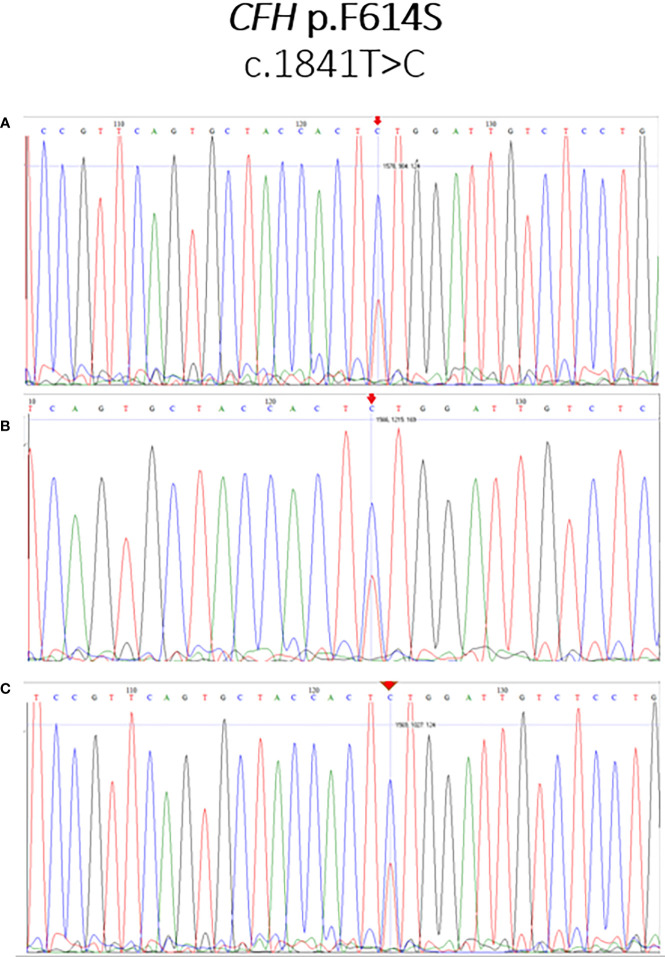
Sanger sequencing validation of the novel variant CFH:c.1841T>C (F614S). The mutation CFH:c.1841T>C (F614S), identified as T/C heterozygote, is marked by an arrow. Illustrative examples for the patients LN21 **(A)**, PI20 **(B)**, and PI22 **(C)** are shown.

Selected cases with enough DNA were sequenced using DyeTerminator Sanger Sequencing on 3730xl DNA Sequencer (Applied Biosystems), and the presence of the mutation was confirmed in all re-sequenced cases. The results of Sanger sequencing are illustrated in **Figure 2**.

### Review of genetic variation in ancestry-matched reference data

Because the variant *CFH*:c.1841T>C *(*F614S) was detected in 70% of the currently studied cases, there is a possibility that this novel variant is population-specific. To address this possibility, we evaluated genetic variation in genes *CFH* and *CD46* in a control cohort of 110 healthy Egyptians, the largest dataset of Egyptian whole-genome sequencing to date. The raw sequencing data at the p.F614S *CFH* variant position were checked. The coverage was found to be sufficient in all, but one individual, to conclude that there is no variant at this position in the reference data and no structural variants overlap it. Furthermore, in the Egyptian reference data, there are no variants in exon 13 of *CFH*, in which this novel variant is located. In the ancestry matched-reference data, 12 of 22 *CFH* coding exons were found to carry overall 15 variants, of which 7 are missense variants. Of those, only one is predicted to be deleterious, rs35453854 in exon 11, observed in a heterozygous state in two Egyptian control individuals, which amounts to an allele frequency of less than 1%. According to the 1000 Genomes project (51), this variant is common in Africans (7%) and rare elsewhere in the world (<=1%); it is also linked to various phenotypes.

**Figure 3:**
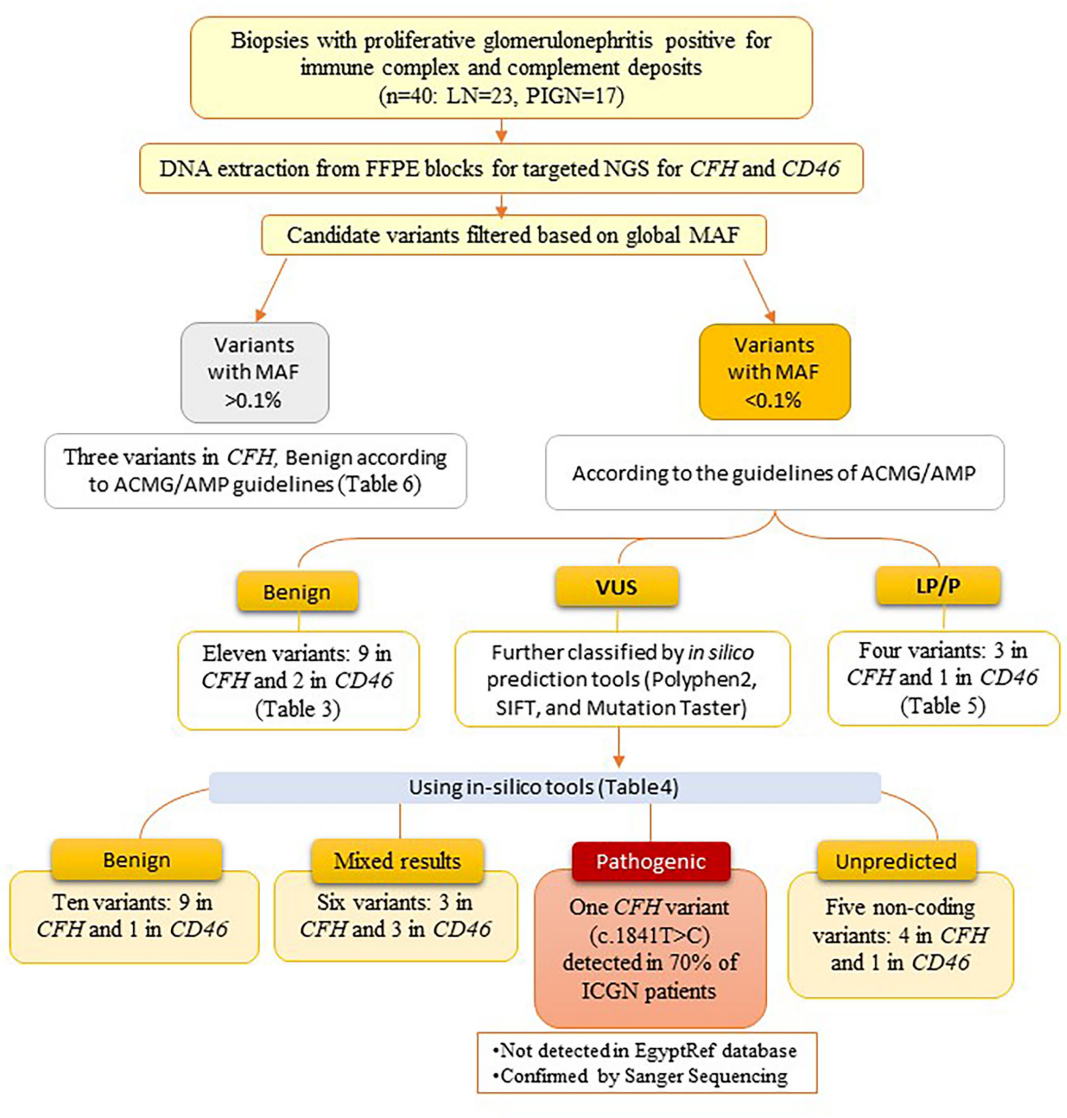
Flowchart showing a summary of the variants detected in CFH and MCP genes among the studied cases.

A summary of the variants detected in *CFH* and *MCP* genes among the studied cases is illustrated in **Figure 3**.

## Discussion

The current study explored, for the first time, the genetic characterization of complement-regulating genes in Egyptian patients with immune complex-mediated GN. Using targeted NGS, The study identified a novel variant (*CFH*:F614S) in the majority of both LN and PIGN cases, that was not found in ancestry matched reference data. To the best of our knowledge, this study is the first of its kind conducted on Egyptian patients.

Among the currently studied LN cases, three pathogenic nonsense mutations in the *CFH* gene were detected in three cases of active proliferative LN (two were children). Although pathogenic mutations in the *CFH* gene are highly associated with aHUS, none of our cases had a history of aHUS. The detected pathogenic mutations involved SCRs 3, 12, and 18. All were heterozygous. Similar mutations have been previously reported in patients with SLE, especially in children with LN. A nonsense mutation involving SCR3 of *CFH* was previously detected in a consanguineous Italian family. The parents were heterozygotes with FH serum levels half that in the normal human serum, and three siblings were homozygous for the mutation with complete deficiency of FH. One of the children had SLE with nephritis that progressed to chronic renal failure (53, 54). Similarly, mutations in SCR18 have been previously reported among patients with SLE. One of them was the mutation *CFH*:c3148A>T (p.N1050Y, SCR18) that was reported among SLE patients in a Swedish study and was significantly associated with earlier onset of LN (55). The stop mutation *CFH*:c.2103G>A (p.W701*, SCR12), that we detected in exon 15 in a female child with LN, was previously reported in a child with Shiga toxin-associated hemolytic uremic syndrome (STEC-HUS) (56). The patient was heterozygous for the mutation and had a quantitative deficiency of serum FH and C3 (56). He was treated with Eculizumab together with antibiotics and meningococcal vaccine and had a favorable outcome.

More than 50 pathogenic and likely pathogenic mutations in *CFH* have been reported in ClinVar (57); of these, thirteen are nonsense (null) variants across ten different exons (57, 58). Mutations in *CFH* are classified into type I and type II (58, 59). Type I mutations are associated with low circulating FH, either partial or complete deficiency and are usually linked to glomerulonephritis and DDD. Type II mutations usually cause protein dysfunction due to defective binding to anionic sites regardless of their serum level. These are usually seen in the terminal regions of *CFH*. Type II mutations are generally associated with aHUS. Null mutations such as those detected among the currently studied cases are considered type I mutations expected to result in premature termination of the protein and, consequently, low systemic FH levels (14).

In the study by Wang et al. (21), a significant association was found between serum FH level and the disease activity and histopathologic parameters of LN. The serum FH level was lowest in classes III and IV-S and was negatively associated with total activity indices, endocapillary hypercellularity and leucocyte infiltration, indicating an anti-correlation between FH serum levels and the activity of renal pathologic injury. They suggested that low FH may be explained by either higher consumption due to overactivation of the complement system in LN, the presence of autoantibodies, or finally, the presence of *CFH* gene mutations (21). Dysfunction of FH (whether due to low serum level or due to poor protein function) will lead to failure of inactivation of alternative pathway c3 convertase (C3bBb) on the cell surfaces, including the glomerular endothelial cells leading to severe, unchecked complement activation, and consequently endothelial injury that results in the proliferative lesions of LN, and occasionally may result in secondary thrombotic microangiopathy (14).

In the current study, one likely pathogenic mutation in the *CD46* gene was detected in one case of LN. It was a missense mutation in exon 5 that encodes for SCR3 (CD46:c.667T>C, p.C223R). It is a novel mutation that was not previously reported. SCR3 affected by this mutation is known to carry ligand (C3b and C4b) binding sites, so it is important for the complement regulatory function of the MCP (60). A similar finding was detected in the study of Jönsen et al. (55), where a likely pathogenic mutation in exon 5 of the *CD46* gene was detected in one SLE patient from the southern Sweden population. In their study, they found that mutations in *CFH* and *CD46* were not as common as in aHUS; however, these mutations were associated with earlier onset of nephritis (55).

Twenty-two variants of uncertain significance were detected in the current study among both LN and PIGN cases. Interestingly, one of these VUS variants (CFH:F614S) was highly enriched among the patients, being detected in about 70% of the studied cases (17 (74%) of LN cases and 11 (65%) of PIGN cases). Sanger sequencing was carried out confirming the presence of this mutation in our cases. It is a novel missense mutation in exon 13 of *CFH* (*CFH*:c1841C>T, p.F614S, SCR10) that was not previously reported, i.e., it was not identified in large reference data sets such as the 1000 Genomes project (51) or gnomAD (52). *In-silico* tools PolyPhen-2, SIFT, and MutationTaster predicted this variant as probably damaging, deleterious or disease-causing, respectively. Other mutations affecting SCR10 of FH have been reported previously, especially in cases with aHUS (61). Functional data about variants in SCR10 are still lacking. Evaluation of genetic variation in the *CFH* gene *in* the largest dataset of Egyptian whole-genome sequencing to date revealed that there is no variant was detected in that position among the studied 110 healthy Egyptians. Although the dataset is based on low coverage sequencing, the coverage was found to be sufficient in all, but one individual, to conclude that this variant is not common in the healthy control group (62). Furthermore, the variant is novel, i.e., has no dbSNP ID yet and has not been identified at all in any individual worldwide. Accordingly, it is very unlikely that this variant is common. Based on the finding that this variant is highly enriched in patients, the possibility of having a potential disease-causing role is raised and needs further investigation. We thus hypothesize that the variant CFH:F614S occurs much more often and perhaps even exclusively in Egyptian patients with LN and postinfectious GN compared to controls, and it might contribute to both diseases *via* a similar molecular mechanism.

Among the seventeen studied cases of post-infectious GN, several variants of uncertain significance have been detected in both studied genes, yet no pathogenic mutations were identified. This may be due to the strict selection criteria, as we excluded cases with sole deposition of C3 to exclude cases of C3 glomerulopathy (C3G) presenting after an episode of infection. However, the presence of variants of uncertain significance in complement regulating genes does not completely exclude the possibility of early C3G. The relationship between cases of post-infectious GN and C3G has been well established. Many studies proved that cases of atypical, a prolonged course of GN usually have an underlying defect in complement regulation due to genetic variants in FH and its related proteins (6, 7, 33, 63, 64).

Three of the common SNVs in the *CFH* gene were detected in our cohort. These variants were in exon 2 (rs800292, p.V62I), exon 7 (rs1061147, A307A), and exon 13 (rs3753396, Q6672Q). At least one of these SNVs was present in 24 (60%) of the patients (15 (65%) of LN and 9 (53%) of post-infectious GN cases). Two of these SNVs were present in combination in two cases. The association of these SNVs with aHUS, age-related macular degeneration, and DDD was studied in several genome-wide association studies (GWAS) (65–73). A large Chinese cohort compared the differences between alleles and genotype frequencies of rs800292. No significant difference was observed among the three studied groups of LN, SLE without nephritis and the control group, so probably it was not linked to the disease process (9). Bonomo et al. (74) performed targeted genotyping for 13 *CFH* variants to study their association with end-stage kidney disease in the African American population. They found a significant association between the SNV rs1061147 and diabetic and non-diabetic end-stage kidney disease (ESKD) and a significant association between SNV rs3753396 and non-diabetic ESKD in African Americans (74).

Implementing genetic testing for complement regulating genes in cases of atypical post-infectious GN and LN is essential to expand our knowledge in this field which will pave the road for the emergence of new, more effective therapies. Recently, drugs targeting the complement system have been suggested for renal diseases. The Identification of the different roles for different complement components in the pathogenesis of GN gave hopes to developing new targeted therapies that may decrease the need for long-term non-specific immunosuppression. Several anti-complement drugs have been approved by Food and Drug Administration (FDA), such as the monoclonal anti-C5 antibody; Eculizumab, and the inhibitor of C1 (C1-INH); Cynrize (75, 76). Pegcetacoplan (C3 inhibitor) was recently approved by the FDA for paroxysmal nocturnal hemoglobinuria, in addition to avacopan (C5a receptor antagonist) for ANCA-associated vasculitis. Such medications may be of potential role in the treatment of GN. New complement inhibitors have been developed and tested in preclinical settings, in addition to the new generation of inhibitors currently being assessed in clinical studies for the treatment of GN. There are various forms of inhibitors; including small proteins (e.g., nomacopan), recombinant proteins (e.g., berinert; C1 esterase inhibitor) that can replace missing or faulty proteins, small interfering RNAs (e.g., ALN-CC5), and humanized monoclonal antibodies (e.g., OMS00620646) (77). Continuing research in the field of the complement system in glomerulonephritis may help introduce new, more effective drugs with fewer side effects.

The limitations to this study are the relatively small number of patients, the lack of factor H serum values, the lack of actual serum complement levels and the lack of segregation data among the relatives of patients harboring the mutations. Also, the sequencing was carried out on only two of the complement-regulating genes. Nevertheless, the study provided informative data regarding the different mutations encountered in *CFH* and *CD46* among cases of ICGN with novel findings, which warrants further functional studies, including serum measures for FH and serum complement levels, to explain the role of these variants in the disease course and predisposition.

In summary, this study describes the genetic variations in two of the complement regulating genes among a group of Egyptian patients with LN or PIGN. Mutations in complement regulating genes *CFH* and *CD46* are not uncommon in Egyptian patients with immune-complex mediated GN. The novel mutation F614S in the *CFH* gene is highly enriched in our patients and thus may be associated with the pathogenesis of ICGN in Egyptian patients. Variants of uncertain significance mandate further studies to delineate their pathogenetic potential and impact on the clinical course and outcome.

## Data availability statement

The original contributions presented in the study are included in the article/**Supplementary Material**. Further inquiries can be directed to the corresponding authors.

## Ethics statement

The studies involving human participants were reviewed and approved by the ethics committees of the Faculty of Medicine, Alexandria University and of the College of Medicine, University of Sharjah.Written informed consent for participation was not required for this study in accordance with the national legislation and the institutional requirements.

## Author contributions

Conceptualization: NB, IT, and RH; methodology: HG, TV, PB, and AM; validation: IT, HA, MS-A, and HB; formal analysis: AB, IW, AN, and RH; resources: NB; data curation: HG, IT, AB, MS-A, and RH; funding acquisition: IT; writing—original draft preparation: HG; writing—review and editing: IT, MS, AE, and SE-G; supervision: IT, RH and NB; All authors contributed to the article and approved the submitted version.

## Funding

This research was funded by the University of Sharjah, grant number 1801090236-P.

## Acknowledgments

The authors acknowledge the support of the University of Sharjah and the Sharjah Institute for Medical Research, University of Sharjah. HB and IW acknowledge the support of the Deutsche Forschungsgemeinschaft (DFG, German Research Foundation) under Germany`s Excellence Strategy – EXC 22167-390884018

## Conflict of interest

The authors declare that the research was conducted in the absence of any commercial or financial relationships that could be construed as a potential conflict of interest.

## Publisher’s note

All claims expressed in this article are solely those of the authors and do not necessarily represent those of their affiliated organizations, or those of the publisher, the editors and the reviewers. Any product that may be evaluated in this article, or claim that may be made by its manufacturer, is not guaranteed or endorsed by the publisher.
