# Supplementary material for: Genetic analysis of CFH and MCP in Egyptian patients with immune-complex proliferative glomerulonephritis
Source: Front Immunol. 2022 Sep 23;13:960068. doi: 10.3389/fimmu.2022.960068 (PMC9539770; doi:10.3389/fimmu.2022.960068)
Supplement: Supplementary file 1 [file DataSheet_1.docx]

Supplementary Material

**Supplementary Tables:**

**Table S1:** Primers used to amplify *CFH* gene.

| Exon | Forward | Reverse | Amplicon Size (bp) |
| --- | --- | --- | --- |
| 1 | **AGGCGGAGCATCTAGTTT** | **CCTGTGAAAAGCATCATTAGCA** | **258** |
| 2 | **GATTGCAATGAACTTCCTCCA** | **CCATTCTCCCTTCCTGCATA** | **156** |
| 2 | **AGAAGGCACCCAGGCTATCT** | **TGGATTAAGAGCAACCCATTC** | **100** |
| 3 | **CTTGTTCCCCCACTCCTAC** | **CCCCTCATTACATGTATACACAGC** | **182** |
| 4 | **TGGACACTCAGAATGGCATC** | **GTCTACCTTCACATATAGG** | **160** |
| 5 | **CAGTTGTGAAGTGTTTACCAGTGA** | **GCCTGAGTTACATACAAACCGTA** | **119** |
| 5 | **TGGAACCAGATCGGGAATAC** | **CCCACACACTTTGGTTTCTCT** | **136** |
| 6 | **TGTCCTGGTCACAGTCCTTT** | **CCAGATTCAGTGCATACAGCA** | **207** |
| 7 | **CGGATACTTATTTCTGCATTAT** | **CATCTCGGAGCAGGTATCCAG** | **213** |
| 8 | **TTGCAAGTGAAACCTTGTGA** | **ACCATCCATCTTGTGTGCAAT** | **181** |
| 8 | **TGGGATCACATTCATTGCAC** | **TGCTCTCCTTTCTTCGATCTTT** | **106** |
| 9 | **TATAGACGTTGCCTGCCATC** | **TGACATAAACATTTTGCCACA** | **208** |
| 9 | **TCATTGTTATGGTCCTTAGGAA** | **GGATGGCAGGCAACGTCTAT** | **211** |
| 11 | **GCAGATGGTGAAACATCAGGA** | **CAGCCCCCACAAAAAGACTA** | **150** |
| 11 | **CCAGGTTTTCAGTTACAAATG** | **CCTGATGTTTCACCATCTGCT** | **246** |
| 12 | **CCCAGTATTTATGAATGCCAGA** | **TGGGTAAATCAGACCAACCA** | **155** |
| 13 | **AAGAAAGAGAATGCGAACTTCC** | **GGAGGTCAGGAGACAATCCA** | **168** |
| 14 | **ATGTGGTCCACCTCCTGAAC** | **TGTCCACTCTCCATCAACAC** | **151** |
| 15 | **TGGAGGAGAGTACCTGTGGAG** | **CACACACTGGGGAAGTTGG** | **179** |
| 16 | **GTTGGTTTGATTCCTATCATTTG** | **CTTCTGGATCCCATCTTCCAT** | **218** |
| 17 | **ACAATTATGCCCACCTCCAC** | **GCCATCTTCCATCTTTGCATGT** | **152** |
| 18 | **CCATGTTCACAACCACCTCA** | **TGGCCTAACCTTCACACTGA** | **187** |
| 19 | **GGCCTTCCTTGTAAATCTCCAC** | **TTATGCATGATGGAGGGTGAGAC** | **175** |
| 20 | **GTATAAGGCGGGTGAGCAAG** | **CTCCCAAAGTGCTGGGATTA** | **199** |
| 20 | **GCTTTGAAAATGCCATACCC** | **ACTGGCTCCATCCATTTTGT** | **104** |
| 21 | **ATCCGCCCACAGTACAAAAT** | **CCTTTGCATTGAGGTGGTTC** | **163** |
| 22 | **TGCTCCAGCTTCATCAGTTG** | **GCATTTTGGTGGTTCTGACC** | **106** |
| 22 | **TGCTCTCACAACAAATCAAGTG** | **TTGATACAAGTTCTGGCATTGG** | **160** |
| 23 | **CCGTGTGTAATATCCCGAGAA** | **GTTCGCAATGTGTGAGAACG** | **152** |
| 23 | **AACGGGGATATCGTCTTTCA** | **GGTGTGCACTTTATGATTGATTC** | **114** |

**Table S2:** Primers used to amplify *CD46* gene.

| Exon | Forward | Reverse | Amplicon size (bp) |
| --- | --- | --- | --- |
| 1 | TCGGTTTCTCTGCTTTCCTC | GGTCCTACCGGAGAAGGAGT | 156 |
| 2 | TCCATGGCTGATGAAAGTGA | AGGCGTCATCTGAGACAGGT | 245 |
| 3 | TTCCCACCCATTCAAAAGAG | AAGGAGCAACTTACCCCTCA | 212 |
| 4 | AGAAACCACCCCCTCAAACT | TCACATATTGGGGGCTTACC | 204 |
| 5 | CCTGGACCAGATCCATTTTC | CAGGAGGAGGAAGCACATACA | 199 |
| 5 | TCCATATTGAATTCACAAACAG | CCAGGTGCAGGATCACAAC | 237 |
| 6 | TTGCATTCCATTCCTTGTCTC | GCTGCCATCGAGGTAAAAAC | 228 |
| 6 | AGCGACACAATTGTCTGTGAC | CAGCAACAACAATAACAAACCAA | 112 |
| 7-8 | TCCTAGTGCTGCCTCCATCT | TATTGCTGGCAATTTCTCCA | 132 |
| 7-8 | GCCAGCAATAACTCCCAAGT | CTAAACCTGAGGCACTGGAC | 107 |
| 9 | TTGATAAGGCCCTGGTGAAT | AAACCAACATGGCACACGTA | 219 |
| 10 | AAGGGATTTTCTACAAAGGTGAA | CCTATGTTTGGGCACCTCAT | 244 |
| 11 | TCAGATGTTTGGGTCATTGC | CGGTTTAACCAATTTACAAGCTG | 155 |
| 12 | TGGAGTTGCAGTAATTTGTGTTG | ACTGAAGCTGCACAAAAGCA | 131 |
| 13 | TCAGCCGTTTTCTCTTCCTC | GCCAATATCTCTTTGCTCAGG | 217 |

**Table S3:** Detailed clinical, laboratory and histopathologic findings of cases of LN.

| Clinical and histologic findings in the LN cases with pathogenetic or likely pathogenic variants | | | | | | | | | | | | | | | | | | | |
| --- | --- | --- | --- | --- | --- | --- | --- | --- | --- | --- | --- | --- | --- | --- | --- | --- | --- | --- | --- |
| Patient | **Age** | **Sex** | **Proteinuria** | **S.Cr** | **S.C3** | **LN Class** | **AI** | **CI** | **Endocapillary hypercellularity** | **Crescents** | **Necrosis** | **Wire loops/hyaline thrombi** | **Neutrophil exudation** | **Interstitial inflammation** | **Sclerotic glomeruli** | **Fibrous crescents** | **IF** | **TA** |  |
| LN12 | 25 | F | 2 | 2 | Low | IV | 11 | 3 | 3 | 1x2 | 1x2 | 2 | 1 | 1 | 1 | 0 | 1 | 1 |  |
| LN7 | 14 | F | 3 | 0.7 | Low | III+V | 8 | 2 | 1 | 1x2 | 1x2 | 1 | 1 | 1 | 1 | 1 | 0 | 0 |  |
| LN19 | 11 | F | 1.8 | 1 | Low | III | 4 | 0 | 1 | 0 | 0 | 1 | 1 | 1 | 0 | 0 | 0 | 0 |  |
| LN6 | 25 | F | 18 | 0.9 | Low | IV+V | 10 | 4 | 2 | 1x2 | 1x2 | 2 | 1 | 1 | 1 | 1 | 1 | 1 |  |
| Clinical and histologic findings in the LN cases with variants of uncertain significance | | | | | | | | | | | | | | | | | | | |
| Patient | **Age** | **Sex** | **Proteinuria** | **S.Cr** | **S.C3** | **LN Class** | **AI** | **CI** | **Endocapillary hypercellularity** | **Crescents** | **Necrosis** | **Wire loops/hyaline thrombi** | **Neutrophil exudation** | **Interstitial inflammation** | **Sclerotic glomeruli** | **Fibrous crescents** | **IF** | **TA** |  |
| LN11 | 22 | 1 | 2 | 3 | Normal | II | 1 | 2 | 0 | 0 | 0 | 0 | 0 | 1 | 0 | 0 | 1 | 1 |  |
| LN13 | 14 | 0 | 1.5 | 1.2 | Normal | II | 0 | 0 | 0 | 0 | 0 | 0 | 0 | 0 | 0 | 0 | 0 | 0 |  |
| LN9 | 23 | 1 | 2 | 0.9 | Low | III | 2 | 2 | 1 | 0 | 0 | 0 | 0 | 1 | 0 | 0 | 1 | 1 |  |
| LN4 | 22 | 0 | 4 | 1.2 | Low | IV | 10 | 3 | 2 | 1x2 | 1x2 | 2 | 1 | 1 | 1 | 0 | 1 | 1 |  |
| LN5 | 40 | 0 | 2.7 | 2 | Low | IV | 8 | 0 | 2 | 1x2 | 1x2 | 1 | 1 | 0 | 0 | 0 | 0 | 0 |  |
| LN20 | 35 | 0 | 1.5 | 0.9 | Low | III | 8 | 1 | 1 | 1x2 | 1x2 | 1 | 1 | 1 | 1 | 0 | 0 | 0 |  |
| LN17 | 18 | 0 | 1.5 | 0.9 | Low | III | 3 | 2 | 1 | 0 | 0 | 1 | 0 | 1 | 0 | 0 | 1 | 1 |  |
| LN16 | 30 | 0 | 2.3 | 0.9 | Low | IV | 9 | 1 | 2 | 1x2 | 1x2 | 1 | 1 | 1 | 1 | 0 | 0 | 0 |  |
| LN3 | 28 | 0 | 3 | 2.5 | Low | IV | 11 | 4 | 3 | 1x2 | 1x2 | 2 | 1 | 1 | 1 | 1 | 1 | 1 |  |

**S.Cr= serum creatinine level (normal range:0.7-1.3 mg/dl ), S.C3= serum C3 level (normal range: 90-180mg/dl)**

**Table S4:** Detailed clinical and histopathologic findings of cases of PIGN.

| Clinical and histologic findings in the PIGN cases with at least one variant of uncertain significance | | | | | | | | | | | | | | |
| --- | --- | --- | --- | --- | --- | --- | --- | --- | --- | --- | --- | --- | --- | --- |
| Patient | **Age** | **Sex** | **Proteinuria** | **S.C3** | **S.Cr** | **Pattern** | **Crescents** | **Necrosis** | **Neutrophil exudation** | **Interstitial inflammation** | **Sclerotic glomeruli** | **IF** | **TA** |  |
| PI19 | 7 | F | 1 | Low | 2.5 | Diffuse endocapillary proliferative | Few | Present | Diffuse | Mild | - | - | - |  |
| PI14 | 14 | M | 0.8 | Low | 0.7 | Mesangial proliferation with few neutrophils | Absent | Absent | Focal | Mild | - | - | - |  |
| PI1 | 12 | F | 2 | Low | 2.7 | Diffuse endocapillary proliferative with crescents | Few | Absent | Diffuse | Mild | - | - | - |  |
| PI6 | 13 | M | 1.5 | Low | 3 | Diffuse endocapillary proliferative with crescents | Few | Absent | Diffuse | Mild | - | - | - |  |
| PI11 | 13 | M | 1.5 | Low | 1.5 | Mesangial proliferation with few neutrophils | Absent | Absent | Focal | Mild | - | - | - |  |
| PI17 | 12 | F | 2.5 | Low | 1.5 | Crescentic GN | Present in >50% of glomeruli | Present | Diffuse | Severe | - | - | - |  |
| PI21 | 7 | M | 1 | Low | 1.5 | Mesangial proliferation with few neutrophils | Absent | Absent | Focal | Mild | - | - | - |  |
| PI23 | 5 | M | 3 | Low | 1 | Diffuse proliferative GN | Absent | Absent | Diffuse | Mild | - | - | - |  |
| PI13 | 13 | M | 0.5 | Low | 0.7 | Mesangial proliferative with few neutrophils | Absent | Absent | Focal | Moderate | - | - | - |  |
| PI8 | 8 | M | 2 | Low | 1.5 | Mesangial proliferation | Absent | Absent | Focal | Mild | - | - | - |  |
| PI20 | 12 | M | 0.7 | Low | 0.7 | Diffuse proliferative GN | Absent | Absent | Diffuse | Moderate | - | - | - |  |
| PI22 | 10 | M | 2 | Low | 3 | Crescentic GN | Present in >50% of glomeruli | Present | Diffuse | Severe | - | - | - |  |
| PI10 | 11 | M | 3 | Low | 3.5 | Crescentic GN | Present in >50% of glomeruli | Absent | Diffuse | Severe | - | - | - |  |
| Clinical and histologic findings in the PIGN cases with only common SNVs | | | | | | | | | | | | | | |
| Patient | **Age** | **Sex** | **Proteinuria** | **S.C3** | **S.Cr** | **Pattern** | **Crescents** | **Necrosis** | **Neutrophil exudation** | **Interstitial inflammation** | **Sclerotic glomeruli** | **IF** | **TA** |  |
| PI16 | 7 | M | 1 | Low | 2.5 | Diffuse proliferative GN | Absent | Absent | Diffuse | Moderate | - | - | - |  |
| PI7 | 6 | M | 1.7 | Low | 1 | Diffuse proliferative GN | Absent | Absent | Diffuse | Severe | - | - | - |  |
| Clinical and histologic findings in the PIGN case with only benign variant | | | | | | | | | | | | | |  |
| Patient | **Age** | **Sex** | **Proteinuria** | **S.C3** | **S.Cr** | **Pattern** | **Crescents** | **Necrosis** | **Neutrophil exudation** | **Interstitial inflammation** | **Sclerotic glomeruli** | **IF** | **TA** |  |
| PI18 | 12 | M | 0.7 | Low | 3 | Crescentic GN | Present in >50% of glomeruli | Present | Diffuse | Severe | - | - | - |  |
| Clinical and histologic findings in the PIGN cases without mutations | | | | | | | | | | | | | |  |
| Patient | **Age** | **Sex** | **Proteinuria** | **S.C3** | **S.Cr** | **Pattern** | **Crescents** | **Necrosis** | **Neutrophil exudation** | **Interstitial inflammation** | **Sclerotic glomeruli** | **IF** | **TA** |  |
| PI5 | 8 | M | 1 | Low | 1.5 | Diffuse proliferative GN | Absent | Absent | Diffuse | Mild | - | - | - |  |

**Table S5.** Allele Frequencies of Benign/Likely benign variants in different populations.

| Gene | Variant* | dbSNP ID | AF (EgyptRef) | AF | AF AFR | AF AMR | AF ASJ | AF EAS | AF SAS | FIN_AF | AF NFE | AF OTH |
| --- | --- | --- | --- | --- | --- | --- | --- | --- | --- | --- | --- | --- |
| *CFH* | 196643015 (T>C) | - | - | - | - | - | - | - | - | - | - | - |
| *CFH* | 196646739 (T>C) | - | - | - | - | - | - | - | - | - | - | - |
| *CFH* | 196654300 (T>C) | - | - | - | - | - | - | - | - | - | - | - |
| *CFH* | 196684820 (T>C) | rs147170171 | - | 0.0000598 | 0 | 0.000029 | 0 | 0 | 0 | 0 | 0.0001147 | 0.0001634 |
| *CFH* | 196684844 (C>T) | - | - | - | - | - | - | - | - | - | - | - |
| *CFH* | 196684855 (T>C) | rs35453854 | 0.009091 | 0.00396 | 0.05315 | 0.00261 | 0 | 0.0008165 | 0.00003266 | 0 | 0.00009711 | 0.002128 |
| *CFH* | 196684859 (G>A) | - | - | - | - | - | - | - | - | - | - | - |
| *CFH* | 196697588 (A>T) | - | - | - | - | - | - | - | - | - | - | - |
| *CFH* | 196712655 (T>C) | rs62641697 | - | 0.00104 | 0.005229 | 0.0003758 | 0.013 | 0 | 0.0001307 | 0 | 0.0001759 | 0.001467 |
| *CD46* | 207925593 (T>C) | - | - | - | - | - | - | - | - | - | - | - |
| *CD46* | 207925626 (C>T) | - | - | - | - | - | - | - | - | - | - | - |

AF: Global (gnomAD: Exomes), AFR: African, AMR: American, ASJ: Ashkenazi Jewish, EAS: East Asian, SAS: South Asian (gnomAD: Exomes).
FIN: European (Finnish), NFE: European (Non-Finish), OTH: Other.

*Chromosomal positions are according to GRCh37

**Table S6:** Allele Frequencies of Variants of uncertain in different populations.

| Gene | Chromosomal position* |  | dbSNP ID | AF (EgyptRef) | AF | AF AFR | AF AMR | AF ASJ | AF EAS | AF SAS | FIN_AF | AF NFE | AF OTH |
| --- | --- | --- | --- | --- | --- | --- | --- | --- | --- | --- | --- | --- | --- |
| *CFH* | 196642242 (G>A) |  | rs747978546 | **-** | 0.0000359 | 0 | 0 | 0 | 0 | 0.0002941 | 0 | 0 | **0** |
| *CFH* | 196643037 (A>G) |  | - | - | - | - | - | - | - | - | - | - | - |
| *CFH* | 196695675 (G>A) |  | - | - | - | - | - | - | - | - | - | - | - |
| *CFH* | 196695705 (A>G) |  | - | - | - | - | - | - | - | - | - | - | - |
| *CFH* | 196695720 (T>C) |  | - | - | - | - | - | - | - | - | - | - | - |
| *CFH* | 196696016 (C>T) |  | - | - | - | - | - | - | - | - | - | - | - |
| *CFH* | 196697572 (G>C) |  | rs767471170 | *-* | 0.00000399 | - | - | - | - | - | - | - | - |
| *CFH* | 196706002 (A>G) |  | - | - | - | - | - | - | - | - | - | - | - |
| *CFH* | 196709801 (G>A) |  | - | - | - | - | - | - | - | - | - | - | - |
| *CD46* | 207958447 (C>T) |  | - | - | - | - | - | - | - | - | - | - | - |
| *CFH* | 196684824 (G>A) |  | rs865963138 | - | - | - | - | - | - | - | - | - | - |
| *CFH* | 196684854 (A>G) |  | - | - | - | - | - | - | - | - | - | - | - |
| *CFH* | 196711088 (G>A) |  | - | - | - | - | - | - | - | - | - | - | - |
| *CD46* | 207925585 (C>T) |  | - | - | - | - | - | - | - | - | - | - | - |
| *CD46* | 207925621 (G>A) |  | - | - | - | - | - | - | - | - | - | - | - |
| *CD46* | 207940529 (A>G) |  | - | - | - | - | - | - | - | - | - | - | - |
| *CFH* | 196694395 (T>C) |  | - | - | - | - | - | - | - | - | - | - | - |
| *CFH* | 196621191 (G>A) |  | - | - | - | - | - | - | - | - | - | - | - |
| *CFH* | 196621330 (T>C) |  | - | - | - | - | - | - | - | - | - | - | - |
| *CFH* | 196645080 (A>G) |  | - | - | - | - | - | - | - | - | - | - | - |
| *CFH* | 196658757 (C>T) |  | - | - | - | - | - | - | - | - | - | - | - |
| *CD46* | 207932924 (T>C) |  | - | - | - | - | - | - | - | - | - | - | - |

EgyptRef: Egyptian Genome Reference, AF: Global (gnomAD : Exomes), AFR: African, AMR: American, ASJ: Ashkenazi Jewish, EAS: East Asian, SAS: South Asian.
FIN: European (Finnish), NFE: European (Non-Finish), OTH: Other.

*Chromosomal positions are according to GRCh37
